# Multi-modality radiomics nomogram based on DCE-MRI and ultrasound images for benign and malignant breast lesion classification

**DOI:** 10.3389/fonc.2022.992509

**Published:** 2022-12-02

**Authors:** Xinmiao Liu, Ji Zhang, Jiejie Zhou, Yun He, Yunyu Xu, Zhenhua Zhang, Guoquan Cao, Haiwei Miao, Zhongwei Chen, Youfan Zhao, Xiance Jin, Meihao Wang

**Affiliations:** ^1^ Department of Radiology, The First Affiliated Hospital of Wenzhou Medical University, Wenzhou, China; ^2^ School of Laboratory Medicine and Life Sciences, Wenzhou Medical University, Wenzhou, China; ^3^ Radiotherapy Center, The First Affiliated Hospital of Wenzhou Medical University, Wenzhou, China; ^4^ Department of Radiology, The Second Affiliated Hospital, Zhejiang University School of Medicine, Hangzhou, China; ^5^ School of Basic Medical Science, Wenzhou Medical University, Wenzhou, China

**Keywords:** breast neoplasms, radiomics, magnetic resonance imaging, ultrasound, strain elastography

## Abstract

**Objective:**

To develop a multi-modality radiomics nomogram based on DCE-MRI, B-mode ultrasound (BMUS) and strain elastography (SE) images for classifying benign and malignant breast lesions.

**Material and Methods:**

In this retrospective study, 345 breast lesions from 305 patients who underwent DCE-MRI, BMUS and SE examinations were randomly divided into training (n = 241) and testing (n = 104) datasets. Radiomics features were extracted from manually contoured images. The inter-class correlation coefficient (ICC), Mann-Whitney U test and the least absolute shrinkage and selection operator (LASSO) regression were applied for feature selection and radiomics signature building. Multivariable logistic regression was used to develop a radiomics nomogram incorporating radiomics signature and clinical factors. The performance of the radiomics nomogram was evaluated by its discrimination, calibration, and clinical usefulness and was compared with BI-RADS classification evaluated by a senior breast radiologist.

**Results:**

The All-Combination radiomics signature derived from the combination of DCE-MRI, BMUS and SE images showed better diagnostic performance than signatures derived from single modality alone, with area under the curves (AUCs) of 0.953 and 0.941 in training and testing datasets, respectively. The multi-modality radiomics nomogram incorporating the All-Combination radiomics signature and age showed excellent discrimination with the highest AUCs of 0.964 and 0.951 in two datasets, respectively, which outperformed all single modality radiomics signatures and BI-RADS classification. Furthermore, the specificity of radiomics nomogram was significantly higher than BI-RADS classification (both *p* < 0.04) with the same sensitivity in both datasets.

**Conclusion:**

The proposed multi-modality radiomics nomogram based on DCE-MRI and ultrasound images has the potential to serve as a non-invasive tool for classifying benign and malignant breast lesions and reduce unnecessary biopsy.

## Introduction

Breast cancer (BC) is the most prevalent cancer and the leading cause of cancer-related death in females worldwide (1, 2). Early detection and diagnosis are the keys to reduce mortality for patients with BC (3). Mammography, ultrasound (US), and magnetic resonance imaging (MRI) are well-known imaging modalities for the detection and differentiation of breast lesions (4–6). Among them, mammography and US are commonly used for breast screening (7, 8). However, limited by the overlap of breast parenchyma, lesions in mammography may be missed or misidentified, particularly in women with dense breast tissue (9). US and MRI, which are two common imaging modalities for the clinical diagnosis of breast tumor besides mammography, can provide different and supplementary information for the same regions of breast lesion (10).

Breast US is an important non-radiation imaging modality to reveal morphological characteristics of breast tumors as an initial examination or to evaluate the suspicious findings at mammography or MRI (8). However, conventional B-mode ultrasound (BMUS) cannot provide spatial information of breast tumors and has been reported with limited accuracy for diagnosis of BC (11, 12). In recent years, US elastography is increasingly used in the diagnosis of BC and included as an important parameter in the Breast Imaging Reporting and Data System (BI-RADS) category (13). Strain elastography (SE) and shear wave elastography (SWE) are two main types of US elastography, in which SE is a semi-quantitative elastographic technique based on the displacement of the tissue and can reflect stiffness of the target lesion. SE plays an important role in differentiating breast lesions based on the fact that malignant lesions are usually stiffer than benign lesions and has the potential to reduce unnecessary biopsy by improving the specificity of breast US (14). Dynamic contrast enhanced MRI (DCE-MRI) has a high spatial resolution with the availability of 3D images and is widely adopted in the clinical diagnosis of BC (15). Although a high sensitivity (85%-97%) of DCE-MRI was achieved in the differentiation of BC, especially for the evaluation of vascular properties, relatively poor and variable specificity (37%-86%) was reported (16–18). Combining US and DCE-MRI to improve the diagnostic performance of BC has been reported (19).

BI-RADS provides a standardized classification system of breast imaging and is widely used in the clinical diagnosis of BC (13). However, visual assessment and subjective reading based on the qualitative descriptions of image features will inevitably lead to large interpretation variability, which only achieved moderate levels of inter-observer agreement (20). In order to tackle these problems, radiomics analysis based on high-throughput quantitative features extracted from radiographic images has been proposed and developed to improve the diagnosis of BC (21). Studies demonstrated that radiomics analysis based on US or DCE-MRI was useful in breast tumor classification (22, 23), prediction of lymph node metastasis (24) and Ki-67 expression levels (25). Multiparametric MRI (26) and dual-model US radiomics (27) have also been investigated, but only one single imaging method was used. To the best of our knowledge, multi-modality radiomics analysis based on the combination of DCE-MRI and US images was rarely reported, especially combined with US elastography. Different imaging methods can make up for the shortcomings of a single modality and the combining of radiomics features extracted from different imaging modalities can build better models based on their complementary information.

The purpose of this study is to develop a multi-modality radiomics nomogram based on DCE-MRI, BMUS and SE images for the classification of benign and malignant breast lesions.

## Materials and methods

### Study population

**Figure 1:**
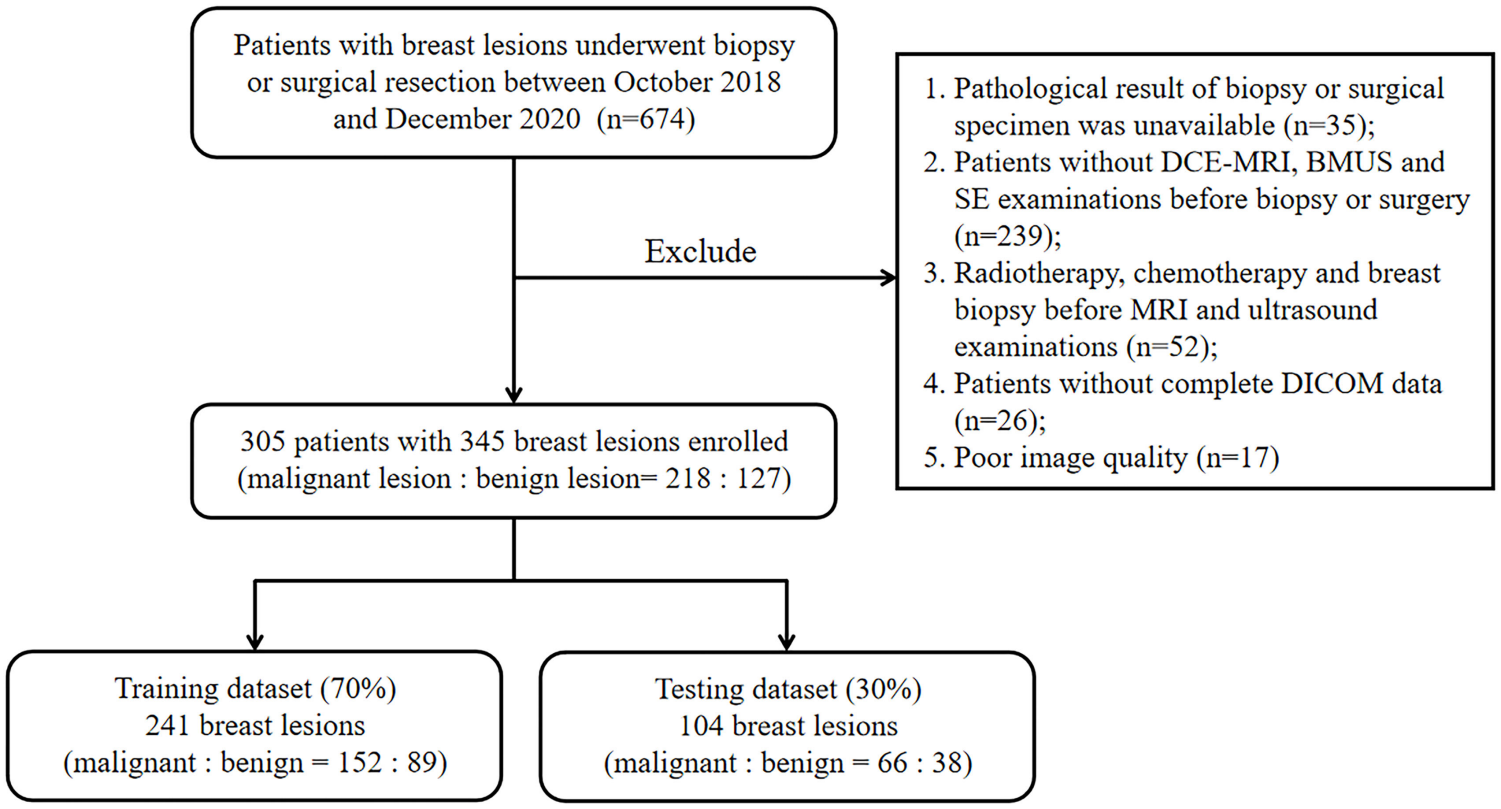
The flowchart of patient selection for this retrospective study.

Patients with breast lesions who underwent biopsy or surgical resection between October 2018 and December 2020 were retrospectively reviewed in this study. This retrospective study was approved by the Ethics Committee in Clinical Research (ECCR) of the authors’ hospitals and informed consent was waived. The exclusion criteria were as follows: (1) pathological result of biopsy or surgical specimen was unavailable for the target lesion; (2) patients without DCE-MRI, BMUS and SE examinations before biopsy or surgery within one month; (3) patients had performed radiotherapy, chemotherapy, or breast biopsy before MRI and ultrasound examinations; (4) patients without complete DICOM data for each examination; (5) patients with poor-quality images. Finally, 345 breast lesions from 305 patients were enrolled in this study. The lesions were randomly divided into training (n = 241) and testing (n = 104) datasets at a ratio of 7:3. The flowchart for patient selection was shown in **Figure 1**.

Conventional clinical characteristics for all patients, including age and primary site, were obtained from electronic medical records. The maximal diameter of the lesion was measured on DCE-MRI image by a radiologist with 16 years of experience in breast disease.

### Image acquisition and tumor segmentation

All patients were scanned in the prone position with a 3.0T MRI scanner (SIGNA HDx, GE Healthcare) using a dedicated 8-channel bilateral breast coil with identical imaging protocol. BMUS and SE were performed using the HI VISION Preirus system (Hitachi Medical, Tokyo, Japan) equipped with a 5-13 MHz linear array transducer. The examinations were performed by two radiologists with 11 and 10 years of experience in breast US. The BMUS was first used to detect and evaluate the lesion, and SE was further performed to access the stiffness of the target lesion. As the SE elastogram was a composite of translucent color elastographic image and the corresponding BMUS image, the elasticity data was reconstructed to generate a purified gray-scale elasticity image for feature extraction (28, 29). The detailed imaging information and post-process for DCE-MRI, BMUS and SE images are provided in **Supplementary Appendix A1**.

**Figure 2:**
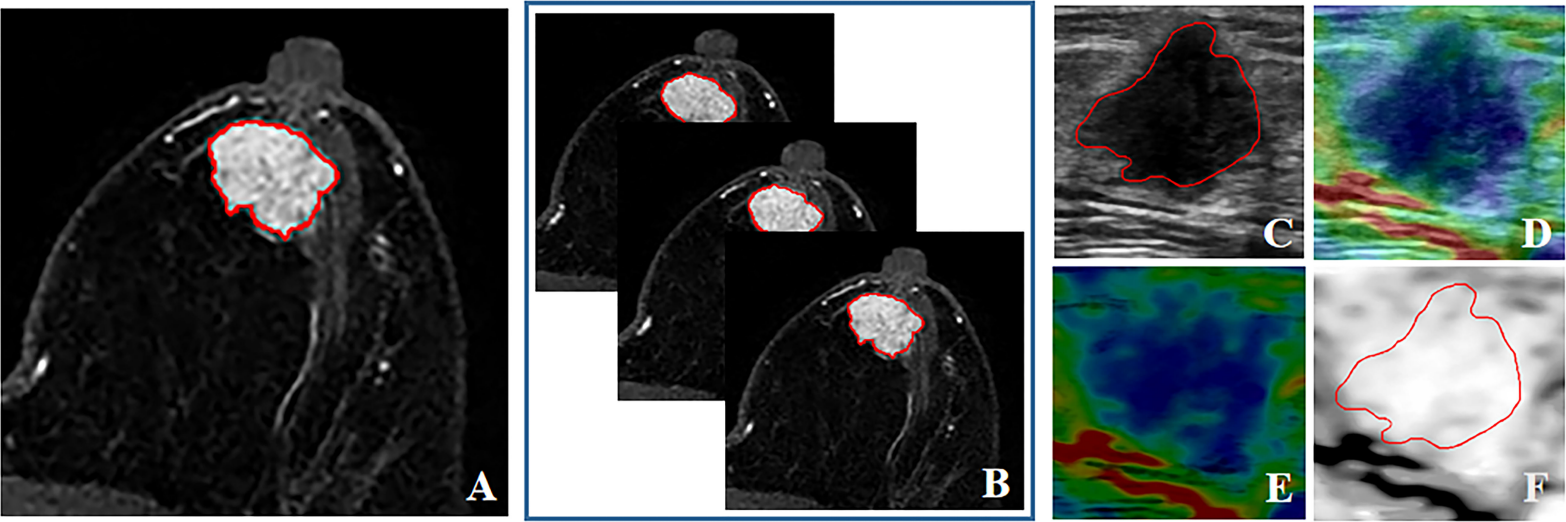
The contours for the same lesion between three imaging modalities of a typical patient. **(A)** DCE-2D; **(B)** DCE-3D; **(C)** BMUS; **(D)** SE elastogram, displayed as a translucent color elastographic image superimposed on the corresponding BMUS image; **(E)** Pure color elasticity image, obtained by subtracting the BMUS from the SE elastogram; **(F)** Pure gray-scale elasticity image, the contour was mapped from BMUS.

The tumors were manually contoured in three imaging modalities by a junior breast radiologist and verified by a senior radiologist using the 3D Slicer software (version 4.11, https://www.slicer.org). To assess the stability of radiomics features, manual segmentation was also performed independently by another radiologist with fifty-five randomly selected lesions to calculate the inter-class correlation coefficient (ICC) of radiomics features (30). Two radiologists were both blinded to the final pathological diagnosis. As the contrast between tumor and background tissue reached a peak in DCE-MRI, the second post-contrast phase of DCE sequence was used for tumor segmentation and radiomics feature extraction (31). Two types of tumor segmentation for DCE-MRI: DCE-2D and DCE-3D, were used to compare their performance of radiomics signatures in this study. DCE-2D and DCE-3D indicated the maximum slice of the tumor and the full slices of the tumor in DCE-MRI, respectively. For US image, the lesion regions were manually delineated on BMUS images and then mapped to the SE images (28). For patients with 2 or more lesions, the lesions with available pathological results were all contoured. Radiomics features were extracted and analyzed for individual lesion. The contours for the same lesion between three imaging modalities of a typical patient were shown in **Figure 2**.

### Radiomics feature extraction

Artificial Intelligence Kit software (A.K, version V3.2.0, GE Healthcare) was used for radiomics feature extraction, according to the reporting guidelines of Image Biomarker Standardisation Initiative (IBSI) (32). Spatial resampling to 1 mm isotropic voxels was applied on DCE-MRI images to standardize for pixel size and slice thickness variations. For DCE-MRI, BMUS and SE images, the bin width of pixel level was set to 25. Seven groups of features were extracted from the tumor regions of the original, Laplacian of Gaussian (LoG), wavelet and local binary pattern (LBP) of each image, respectively: first order, shape, gray-level co-occurrence matrix (GLCM), gray-level size zone matrix (GLSZM), gray-level run length matrix (GLRLM), neighboring gray tone difference matrix (NGTDM) and gray-level dependence matrix (GLDM).

### Radiomics feature selection and signature construction

The data were randomly divided into training (70%) and testing datasets (30%) based on lesions. A three-step feature selection methodology was designed to reduce the data dimension and select key features for signature construction in the training dataset. The ICCs were first used to estimate the reproducibility and stability of radiomics features. Radiomics features with ICCs greater than 0.90 were selected. Then Mann-Whitney U tests were employed to select radiomics features with *p* < 0.05 as potentially robust features. Finally, the least absolute shrinkage and selection operator (LASSO) was applied to further select key features by elastic net parameter tuning and 10-fold cross-validation.

The selected features were combined linearly with relative weights to calculate the radiomics score (Rad-score) and construct the single modality radiomics signature for each lesion. For the combination of multiple imaging modalities, all the selected key features of corresponding images were combined and introduced into logistic regression to construct the radiomics signature. The Rad-score of the multi-modality radiomics signature was obtained *via* a linear combination of all selected features weighted by their corresponding coefficients of the logistic regression. The performance of radiomics signatures were evaluated by the receiver operator characteristic (ROC) curve and area under the curve (AUC) in both training and testing datasets. The corresponding accuracy, sensitivity and specificity were calculated.

### Development, performance, and validation of radiomics nomogram

In the training dataset, univariate logistic regression analysis was first applied to identify potential predictors associated with BC, such as clinical characteristics and radiomics signature. Then, the multivariate logistic regression analysis was used to select independent predictors to develop a radiomics nomogram for benign and malignant breast lesion classification. Nomograms are widely used for diagnosis of malignancy and cancer prognosis, primarily because of their ability to generate an individual probability of a clinical event by integrating diverse prognostic and determinant variables, which meet our desire for clinically integrated models (33). User-friendly graphical interfaces facilitate the use of nomograms during clinical encounters to aid clinical decision making. The discrimination performance of the radiomics nomogram was quantitatively evaluated using ROC analysis and AUC. The calibration of the nomogram was assessed using the calibration curve and the Hosmer-Lemeshow test. The diagnostic performance of the established nomogram was further tested in the testing dataset.

### Radiological evaluation and clinical use

A senior radiologist (with 26 years of experience) who was blinded to the pathological results reviewed the MRI and US images of each lesion by following the ACR BI-RADS 5th edition (13). Then, the BI-RADS category of 345 lesions was classified into 2, 3, 4A, 4B, 4C, and 5 with the combination of MRI and US evaluation (34).

To evaluate the clinical usefulness of the radiomics nomogram, a decision curve analysis (DCA) was performed by guiding biopsy at different threshold probabilities in the testing dataset (35). Moreover, the DCA was also used to compare the additional value of radiomics nomogram relative to BI-RADS classification.

### Statistical analysis

Statistical analysis was performed with R software (version 4.1.0, http://www.R-project.org), SPSS software (version 19.0, IBM, Armonk, NY, USA), and MedCalc software (version 19.8, Mariakerke, Belgium). The Chi-Squared test or Fisher exact test and the Student t-test or Mann-Whitney U test were used to compare categorical variables and continuous variables, respectively. The ICC and LASSO regression was performed using the “irr” and “glmnet” packages. The nomogram and DCA were plotted by the “rms” and “rmda” packages. The MedCalc software was used to perform the ROC curves. Delong test was performed to compare different AUCs. A value of *p* < 0.05 was considered as statistically significant.

## Results

### Patients and lesions

A total of 345 breast lesions in 305 patients (mean age 47.9 ± 10.1 years, range 22-78 years) were enrolled in this retrospective study. The characteristics and pathological information of breast lesions from corresponding patients were shown in **Table 1** and **Supplementary Tables S1**, **S2**. Statistical differences were found between malignant group and benign group in age, maximal diameter and BI-RADS category in both datasets (*p* < 0.05). The characteristics were well balanced between the training and testing datasets.

**Table 1:** Characteristics of breast lesions from corresponding patients in the training and testing datasets.

Characteristics	Training dataset (n = 241)		*p* value	Testing dataset (n = 104)		*p* value
	Malignant (n = 152)	Benign (n = 89)		Malignant (n = 66)	Benign (n = 38)	
Age (years)						
Mean ± SD	49.7 ± 9.9	44.7 ± 9.1	<0.001	50.3 ± 9.7	44.3 ± 11.5	0.006
Range	22–72	24–78		34–68	23–72	
Maximal diameter (cm)						
Mean ± SD	2.3 ± 1.3	1.6 ± 1.2	<0.001	2.5 ± 1.2	1.9 ± 1.4	0.022
Range	0.5–7.8	0.3–6.5		0.7–6.3	0.5–6.5	
Primary site			0.482			0.712
Left	72 (47.4%)	38 (42.7%)		34 (51.5%)	21 (55.3%)	
Right	80 (52.6%)	51 (57.3%)		32 (48.5%)	17 (44.7%)	
BI-RADS category			<0.001			<0.001
2–4A	16 (10.5%)	72 (80.9%)		6 (9.1%)	27 (71.1%)	
4B–5	136 (89.5%)	17 (19.1%)		60 (90.9%)	11 (28.9%)	

BI-RADS, Breast Imaging Reporting and Data System.

### Feature selection and radiomics signature construction

A total of 1316 radiomics features were extracted from DCE-2D and DCE-3D regions of DCE-MRI, respectively. For BMUS and SE images, a total of 1130 radiomics features were extracted from the region of target lesion, respectively. There were 767, 863, 831 and 854 radiomics features with high stability and reproducibility (ICCs > 0.9) for DCE-2D, DCE-3D, BMUS and SE images, respectively. Subsequently, according to the Mann-Whitney U test with a *p* < 0.05, there were 555, 643, 379 and 592 features remained from DCE-2D, DCE-3D, BMUS and SE images, respectively. Based on the LASSO logistic regression, in which 10-fold cross-validation was applied, 8, 18, 8 and 7 radiomics features were screened out to build radiomics signatures from DCE-2D, DCE-3D, BMUS and SE images, respectively **(** **Supplementary Figure S1** **)**. The selected features from corresponding images were combined linearly with relative weights to calculate the Rad-score and construct the radiomics signature for each lesion. The Rad-score calculation formula of each radiomics signature was presented in **Supplementary Appendix A2**.

### Performance of radiomics signatures based on single imaging modality

**Table 2** and **Supplementary Figure S2** showed the detailed diagnostic performance and ROC curve analysis of single modality radiomics signatures. The AUCs comparison between DCE-2D and DCE-3D radiomics signatures in the training and testing datasets were 0.801 vs. 0.877 and 0.782 vs. 0.810, respectively. The DCE-3D radiomics signature showed better diagnostic performance than DCE-2D radiomics signature according to all evaluation metrics in both datasets. Therefore, the selected features of DCE-3D radiomics signature were used to construct the multi-modality radiomics signatures. In addition, the radiomics signatures derived from BMUS and SE images achieved AUCs of 0.819, 0.785 and 0.880, 0.866 in the training and testing datasets, respectively.

**Table 2:** The diagnostic performance of single modality radiomics signatures.

Radiomics signatures	Training dataset				Testing dataset			
	AUC	ACC,%	SEN,%	SPE,%	AUC	ACC,%	SEN,%	SPE,%
DCE-2D	0.801 (0.745–0.850)	77.2 (71.4–82.3)	81.6 (74.5–87.4)	69.7 (59.0–79.0)	0.782 (0.690–0.857)	74.0 (64.5–82.1)	84.9 (73.9–92.5)	55.3 (38.3–71.4)
DCE-3D	0.877 (0.828–0.915)	81.7 (76.3–86.4)	82.9 (76.0–88.5)	79.8 (69.9–87.6)	0.810 (0.722–0.880)	78.8 (69.7–86.2)	89.4 (79.4–95.6)	60.5 (43.4–76.0)
SE	0.880 (0.832–0.918)	80.9 (75.4–85.7)	78.9 (71.6–85.1)	84.3 (75.0–91.1)	0.866 (0.785–0.925)	78.8 (69.7–86.2)	78.8 (67.0–87.9)	78.9 (62.7–90.4)
BMUS	0.819 (0.765–0.866)	75.5 (69.6–80.8)	72.4 (64.5–79.3)	80.9 (71.2–88.5)	0.785 (0.693–0.859)	70.2 (60.4–78.8)	69.7 (57.1–80.4)	71.1 (54.1–84.6)

Data in parentheses are 95% confidence intervals.

DCE, dynamic contrast enhanced; SE, strain elastography; BMUS, B-Mode ultrasound; AUC, area under the curve; ACC, accuracy; SEN, sensitivity; SPE, specificity.

### Performance of radiomics signatures based on multiple imaging modalities

The detailed diagnostic performance and ROC curve analysis of four established multi-modality radiomics signatures were shown in **Table 3** and **Supplementary Figure S3**. The AUCs of four multi-modality radiomics signatures (BMUS + SE, DCE-3D + BMUS, DCE-3D + SE and All-Combination) were 0.895, 0.929, 0.945 and 0.953 in the training dataset, and then confirmed in the testing dataset with the AUCs of 0.874, 0.862, 0.935 and 0.941, respectively. The All-Combination radiomics signature based on DCE-3D, BMUS and SE images achieved the best AUC of 0.941 in the testing dataset with accuracy, sensitivity and specificity of 85.6%, 87.9% and 81.6%, respectively. Rad-score of the All-Combination radiomics signature for each lesion in the training and testing datasets was shown in **Supplementary Figure S4** and the optimal cutoff value was determined to be 0.797. The All-Combination radiomics signature was introduced into the multivariate logistic regression analysis to construct the radiomics nomogram.

**Table 3:** The diagnostic performance of multi-modality radiomics signatures.

Radiomics signatures	Training dataset				Testing dataset			
	AUC	ACC,%	SEN,%	SPE,%	AUC	ACC,%	SEN,%	SPE,%
BMUS + SE	0.895 (0.849–0.931)	83.0 (77.6–87.5)	80.9 (73.8–86.8)	86.5 (77.6–92.8)	0.874 (0.795–0.931)	77.9 (68.7–85.4)	77.3 (65.3–86.7)	79.0 (62.7–90.4)
DCE-3D + BMUS	0.929 (0.889–0.958)	85.5 (80.4–89.7)	86.2 (79.7–91.2)	84.3 (75.0–91.1)	0.862 (0.781–0.922)	79.8 (70.8–87.0)	86.4 (75.7–93.6)	68.4 (51.3–82.5)
DCE-3D + SE	0.945 (0.909–0.971)	89.6 (85.1–93.2)	92.1 (86.6–95.9)	85.4 (76.3–92.0)	0.935 (0.870–0.974)	85.6 (77.3–91.7)	90.9 (81.3–96.6)	76.3 (59.8–88.6)
All-Combination	0.953 (0.918–0.976)	89.2 (84.6–92.8)	89.5 (83.5–93.9)	88.8 (80.3–94.5)	0.941 (0.877–0.978)	85.6 (77.3–91.7)	87.9 (77.5–94.6)	81.6 (65.7–92.3)

Data in parentheses are 95% confidence intervals.

DCE, dynamic contrast enhanced; SE, strain elastography; BMUS, B-Mode ultrasound; AUC, area under the curve; ACC, accuracy; SEN, sensitivity; SPE, specificity.

### Construction and validation of the radiomics nomogram

**Figure 3:**
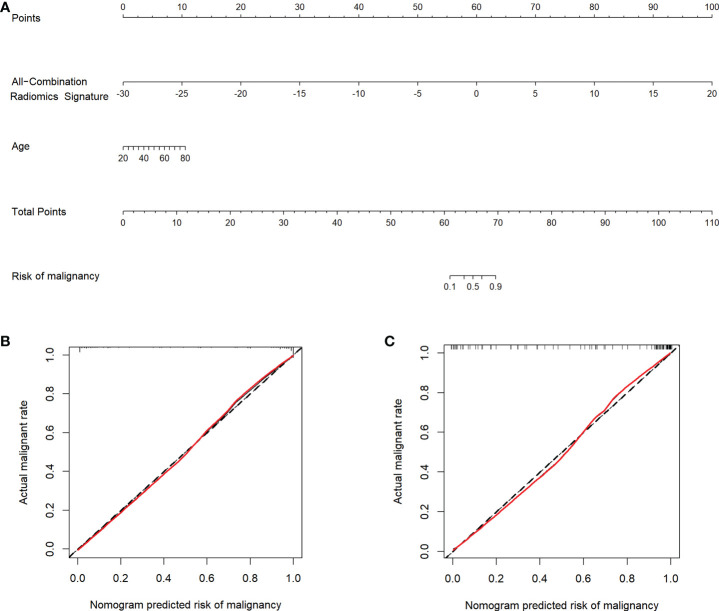
Radiomics nomogram and calibration curves. **(A)** Radiomics nomogram developed in the training dataset incorporates the All-Combination radiomics signature and age. Calibration curves of the radiomics nomogram in the training **(B)** and testing **(C)** datasets.

Multivariate logistic regression analysis showed that the All-Combination radiomics signature and age were identified as independent predictors for benign and malignant breast lesion classification **(** **Table 4** **)**. A multi-modality radiomics nomogram incorporating the All-Combination radiomics signature and age was developed **(** **Figure 3A** **)**. The calibration curve for the radiomics nomogram was tested using Hosmer-Lemeshow test, and yielded a non-significant result (both *p* > 0.1 in training and testing datasets) providing evidence of good calibration **(** **Figures 3B, C** **)**. The radiomics nomogram achieved an AUC of 0.964 in the training dataset with accuracy, sensitivity and specificity of 90.9%, 89.5% and 93.3%, respectively **(** **Table 5** **)**. In the testing dataset, it also showed excellent diagnostic performance, with the AUC, accuracy, sensitivity and specificity of 0.951, 90.4%, 90.9% and 89.5%, respectively. As shown in **Supplementary Table S3**, the AUCs of radiomics nomogram were significantly higher than all single modality radiomics signatures in both datasets (all *p* < 0.03).

**Table 4:** Risk factors associated with malignancy of breast lesions using univariate and multivariate analysis.

Variables	Univariate analysis			Multivariate analysis		
	β	Odds ratio (95% CI)	*p*	β	Odds ratio (95% CI)	*p*
Age	0.055	1.056 (1.026–1.088)	<0.001	0.091	1.095 (1.036–1.158)	0.001
Maximal diameter	0.614	1.847 (1.384–2.465)	<0.001	0.052	1.054 (0.648–1.715)	0.833
Primary site	–0.189	0.828 (0.489–1.402)	0.482			
All-Combination radiomics signature	0.984	2.676 (2.044–3.504)	<0.001	1.023	2.782 (2.059–3.759)	<0.001

**Table 5:** The diagnostic performance of radiomics nomogram and BI-RADS classification.

Metrics	Radiomics nomogram		BI-RADS	
	Training dataset	Testing dataset	Training dataset	Testing dataset
AUC	0.964 [0.932–0.984]	0.951 [0.890–0.983]	0.910 [0.867–0.943]	0.909 [0.837–0.957]
Accuracy^†^	90.9 (219/241) [86.5–94.2]	90.4 (94/104) [83.0–95.3]	86.3 (208/241) [81.3–90.4]	83.7 (87/104) [75.1–90.2]
Sensitivity^†^	89.5 (136/152) [83.5–93.9]	90.9 (60/66) [81.3–96.6]	89.5 (136/152) [83.5–93.9]	90.9 (60/66) [81.3–96.6]
Specificity^†^	93.3 (83/89) [85.9–97.5]	89.5 (34/38) [75.2–97.1]	80.9 (72/89) [71.2–88.5]	71.1 (27/38) [54.1–84.6]
PPV^†^	95.8 (136/142) [91.3–98.0]	93.8 (60/64) [85.5–97.4]	88.9 (136/153) [83.9–92.5]	84.5 (60/71) [76.7–90.0]
NPV^†^	83.8 (83/99) [76.5–89.2]	85.0 (34/40) [72.4–92.5]	81.8 (72/88) [73.7–87.9]	81.8 (27/33) [67.1–90.8]

^†^Data are percentages, with numerator/denominator in parentheses, and 95% confidence intervals in brackets.

AUC, area under the curve; PPV, positive predictive value; NPV, negative predictive value; BI-RADS, Breast Imaging Reporting and Data System.

### Comparison of radiomics nomogram and BI-RADS classification

**Figure 4:**
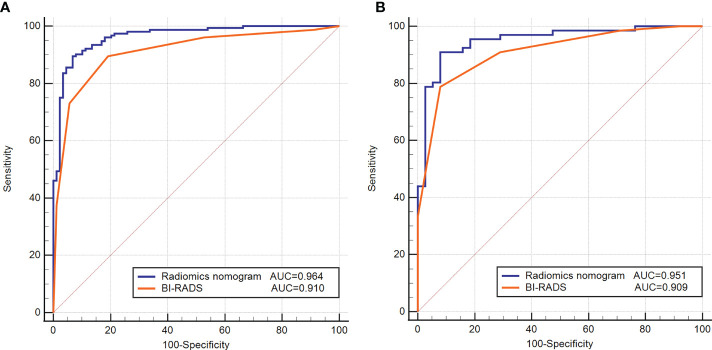
Receiver operating characteristic (ROC) curves of the radiomics nomogram and BI-RADS classification in the training **(A)** and testing **(B)** datasets.

The detailed diagnostic performance and ROC curve analysis between radiomics nomogram and BI-RADS classification were shown in **Table 5** and **Figure 4**. Compared with the BI-RADS classification evaluated by a senior breast radiologist, the radiomics nomogram achieved a significantly higher AUC in the training dataset (0.964 vs. 0.910, *p* = 0.01) and a comparable AUC in the testing dataset (0.951 vs. 0.909, *p* = 0.16). Besides, the accuracy of the radiomics nomogram was better than BI-RADS classification in both datasets. It’s worth noting that the specificity of radiomics nomogram was significantly higher than BI-RADS classification in the training dataset (93.3% vs. 80.9%, *p* = 0.03) and testing dataset (89.5% vs. 71.1%, *p* = 0.04) with the same sensitivity.

### Clinical use

**Figure 5:**
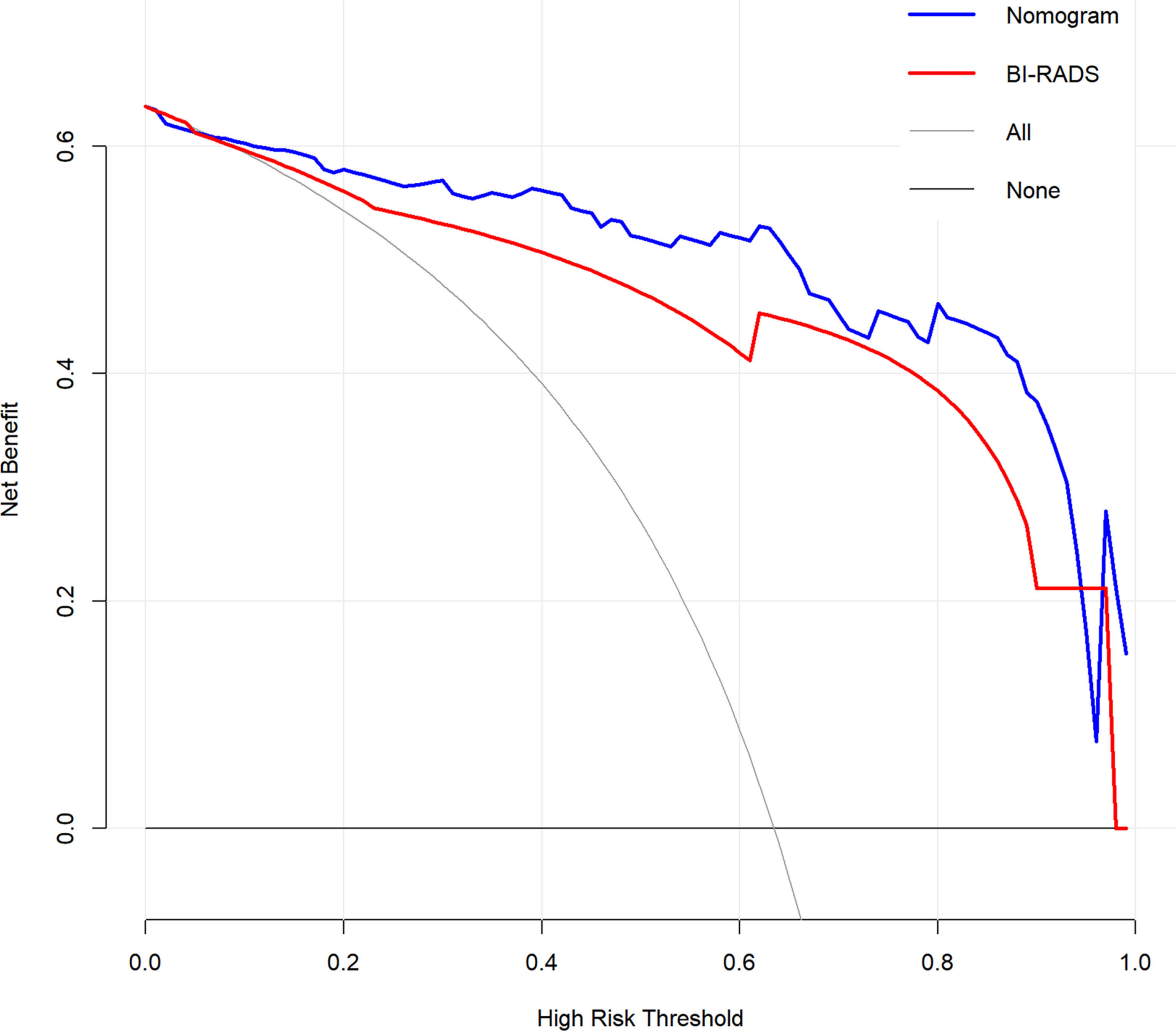
Decision curve analysis (DCA) for the radiomics nomogram and BI-RADS classification in the testing dataset. The y-axis represents the net benefit.

The DCA for the radiomics nomogram and BI-RADS classification were presented in **Figure 5**. The DCA showed that if the threshold probability was more than 5%, the application of radiomics nomogram could provide a better net benefit than BI-RADS classification, none-biopsy scheme and all-biopsy scheme.

## Discussion

In this study, we developed a multi-modality radiomics nomogram incorporating the All-Combination radiomics signature and age for classifying benign and malignant breast lesions. While multiple studies have reported the radiomics analysis developed using MRI (26, 36) or US images (22, 28), the multi-modality analysis was rarely reported. The proposed radiomics nomogram demonstrated excellent diagnostic performance with the highest AUC and accuracy of 0.964, 0.951 and 90.9%, 90.4% in the training and testing datasets, respectively, which outperformed all single modality radiomics signatures and BI-RADS classification evaluated by a senior breast radiologist.

Radiomics based on DCE-MRI has been proven to be useful in the classification of benign and malignant breast lesions (23). Ji et al. used support-vector machine (SVM) to classify breast lesions and achieved an AUC of 0.89 on the independent set (36). It’s notable that the proportion of malignant and benign cases in our study (1.7:1) is more balanced compared to the study of Ji et al. (3:1). Besides, multiple lesions from the same patient in the study of Ji et al. may led to the intra-patient dependence, which was similar to our research. The global analysis of two model selection procedures (LASSO and stepwise models) was proposed for data with intra-patient dependence to best define the set of radiomics features (37). Moreover, the robustness of radiomics features was also important in radiomics analysis and several studies have investigated feature robustness using novel approaches recently (38, 39). In this study, radiomics features with ICC greater than 0.90 were selected as highly robust features. In our research, we further compared the performance of radiomics analysis based on 3D tumor volume and 2D segmentation on the maximum slice of the tumor. The result demonstrated that the DCE-3D radiomics signature showed better diagnostic efficacy and outperformed DCE-2D radiomics signature according to all evaluation metrics. This observation was consistent with the previously reported study, suggesting that the additional benefit may be obtained from using 3D tumor volume, which can better depict spatial heterogeneity (40). However, a recent study indicated that the 3D radiomics analysis showed a similar performance to 2D analysis in predicting axillary lymph node metastasis of BC (41). These studies demonstrated that the effects of 2D or 3D radiomics analysis do not reach a consensus and further studies are required.

Multiple studies have reported that radiomics based on US image can be a useful clinical tool for classifying breast lesions. Romeo et al. proposed a radiomics analysis combined with machine learning to classify breast lesions on BMUS images and achieved an AUC of 0.82, which was similar to our result (22). Additionally, radiomics features extracted from elastography have also been investigated for breast tumor differentiation, achieving an AUC of 0.917 using SE image (28), which was slightly higher than our result of 0.866. The difference may be mainly due to the advanced sonoelastomics method (Cluster-Fv) proposed in the study of Zhang et al, which makes great contributions to model building. Moreover, an automatic image segmentation method using the Chan-Vese level sets was applied in the study of Zhang et al. Recently, several automated segmentation methods have been developed for DCE-MRI and US images, and have the potential to be applied in radiomics analysis (42, 43). However, the manual segmentation by experienced experts is often regarded as the “ground truth” or “gold standard” despite high inter-reader variability (44, 45). It is interesting to note that the AUC of SE radiomics signature in our study was higher than that of BMUS radiomics signature, which is consistent with previous research (46). This finding demonstrated that radiomics features extracted from elastography may be the more valuable predictors for breast lesion classification.

Combination of radiomics features extracted from different imaging modalities has been researched for improving the diagnosis of breast cancer based on their complementary information (26, 47). A multimodal US-based radiomics with attribute bagging was developed using the approach of eight-fold cross-validation and achieved an AUC of 0.919 (47). The cross-validation and bootstrapping methods have been investigated to mitigate the lack of independent external testing set in radiomics analysis recently (48, 49). In our study, we further investigated the value of combining DCE-MRI, BMUS and SE images for the diagnosis of breast lesions using a radiomics approach. Among four multi-modality radiomics signatures, the All-Combination radiomics signature based on DCE-3D, BMUS and SE images showed the best diagnostic performance with an AUC of 0.953 and 0.941 in training and testing datasets, respectively. Interestingly, we observed that the performance of radiomics signature derived from the combination of DCE-3D and SE images is comparable to the All-Combination radiomics signature with a similarly AUC value, demonstrating that DCE-MRI and SE imaging modalities may contribute most to the classification of breast lesions. In this study, the All-Combination radiomics signature and age were identified as independent predictors for benign and malignant breast lesion classification. Older women had a higher probability of malignancy, as has been reported in previous studies (50, 51).

The main contribution of this work is that we developed a multi-modality radiomics nomogram incorporating the All-Combination radiomics signature and age, showing an excellent diagnostic performance in breast lesion classification. The radiomics nomogram achieved AUCs of 0.964 and 0.951 in training and testing datasets, respectively. According to our best knowledge, this is the first study combining MRI and US elastography images for radiomics nomogram building. A dual-model US radiomics nomogram was developed based on shear wave elastography (SWE) and BMUS with performance comparable to BI-RADS classification. The nomogram achieved an AUC of 0.92 which was slightly lower than our result (27). A multimodal classifier combining mammography and DCE-MRI achieved a better diagnostic performance than any single modality model and was in line with our result (52). Recently, Qiao et al. built a MUM-Net classifier based on DCE-MRI and conventional US, which achieved AUCs of 0.858, 0.870 and 0.857 for predicting lymph node metastasis, histological grades, and Ki-67 expression levels, respectively (53). Although the tasks were different and could not be directly compared with our study, they can serve as a reference for the performance of our model. Their result was comparable to our radiomics signature derived from the combination of DCE-MRI and BMUS images with an AUC of 0.862 for breast lesion classification. It’s notable that when the radiomics features of SE images were added to the multi-modality radiomics signature in our study, the AUC was increased to 0.941, indicating the importance of SE imaging modality.

Furthermore, compared with the BI-RADS classification evaluated by a senior breast radiologist, the radiomics nomogram showed better diagnostic performance in both training and testing datasets. It is worth noting that the specificity of radiomics nomogram was significantly higher than BI-RADS classification in both datasets (both *p* < 0.04) with the same sensitivity. The correct benign diagnosis is helpful to avoid unnecessary biopsy. DCA demonstrated that the nomogram could improve breast lesion management non-invasively. Overall, the proposed radiomics nomogram was expected to assist the diagnosis of radiologists and could be possibly implemented in routine clinical practice.

This study had several limitations. First, this was a retrospective study and the included patients were from one institution. Future, the application of cross-validation and bootstrapping approaches may alleviate this question and multicenter studies are necessary to verify the reliability of developed radiomics nomogram. Second, tumor segmentation was performed manually and lacked reproducibility. Thus, we selected radiomics features with ICC > 0.9 to address this problem. An automated and stable segmentation method should be developed in the future. Third, the type of ultrasound elastography we used was SE imaging modality. However, it was reported that SWE is more reproducible and less operator-dependent than SE. The combination of DCE-MRI and SWE radiomics features for classifying breast lesions is in our future research directions. Fourth, carbohydrate antigen 153 (CA153) was not included for analysis due to the lack of available data on some patients in this study, which is a commonly used blood marker for breast cancer diagnosis and management. Furthermore, for MRI images, only the DCE-MRI was included in radiomics analysis, and other sequences such as DWI and T2WI were not analyzed.

In the future, the standardization of DCE-MRI and US imaging between different institutions should be first established. Then a multicenter study should be conducted to verify the generalizability of the proposed nomogram. Further improvements of nomogram could be achieved by merging larger independent datasets from different institutions. Moreover, multiparametric MRI and mammography could be included to enrich the imaging modalities of radiomics analysis. Future work should also include more meaningful clinical characteristics, such as CA153, to further improve the diagnostic performance of radiomics model and finally apply the proposed nomogram to clinical practice.

In conclusion, the multi-modality radiomics nomogram incorporating the All-Combination radiomics signature and age showed better diagnostic performance than all single modality radiomics signatures and BI-RADS classification, which may serve as a non-invasive tool for classifying benign and malignant breast lesions and reduce unnecessary biopsy.

## Data availability statement

The raw data supporting the conclusions of this article are available from the corresponding author on reasonable request.

## Ethics statement

The studies involving human participants were reviewed and approved by Ethics Committee of The First Affiliated Hospital of Wenzhou Medical University. Written informed consent for participation was not required for this study in accordance with the national legislation and the institutional requirements.

## Author contributions

XL, MW, JZha, and XJ conceptualized and designed the study. JZho, HM, ZC and YZ provided the study materials or patients. XL, JZho, YH and ZZ collected and assembled the data. XL, JZha, YX and GC analyzed and interpreted the data. XL, JZha, XJ and MW coordinated, drafted, revised and finalized the manuscript. All authors contributed to the article and approved the submitted version.

## Funding

This work was supported by the Key Laboratory of Intelligent Medical Imaging of Wenzhou (No. 2021HZSY0057), Key Laboratory of Alzheimer’s Disease of Zhejiang Province, Institute of Aging, Wenzhou Medical University, Wenzhou, Zhejiang, China.

## Conflict of interest

The authors declare that the research was conducted in the absence of any commercial or financial relationships that could be construed as a potential conflict of interest.

## Publisher’s note

All claims expressed in this article are solely those of the authors and do not necessarily represent those of their affiliated organizations, or those of the publisher, the editors and the reviewers. Any product that may be evaluated in this article, or claim that may be made by its manufacturer, is not guaranteed or endorsed by the publisher.

## Abbreviations

AUC, Area under the curve; BI-RADS, Breast Imaging Report and Data System; BMUS, B-mode ultrasound; DCA, Decision curve analysis; DCE, Dynamic contrast enhanced; DICOM, Digital Imaging and Communications in Medicine; GLCM, Gray-level co-occurrence matrix; GLDM, Gray-level dependence matrix; GLRLM, Gray-level run length matrix; GLSZM, Gray-level size zone matrix; ICC, Inter-class correlation coefficient; LASSO, Least absolute shrinkage and selection operator; LBP, Local binary pattern; LoG, Laplacian of Gaussian; MRI, Magnetic resonance imaging; NGTDM, Neighboring gray tone difference matrix; ROC, Receiver operating characteristic; ROI, Region of interest; SE, Strain elastography; SWE, Shear wave elastography.
