# Supplementary material for: Multi-modality radiomics nomogram based on DCE-MRI and ultrasound images for benign and malignant breast lesion classification
Source: Front Oncol. 2022 Dec 2;12:992509. doi: 10.3389/fonc.2022.992509 (PMC9755840; doi:10.3389/fonc.2022.992509)
Supplement: Supplementary file 1 [file DataSheet_1.docx]

**Supplementary Material**

**Supplementary Appendix A1** Detailed imaging information and post-process for DCE-MRI，BMUS and SE images

**Ⅰ. Detailed parameters of DCE-MRI sequence**

All patients performed MRI on a 3T scanner (SIGNA HDx, GE Healthcare) using a dedicated 8-channel bilateral breast coil. DCE-MRI was acquired using the volume imaging for breast assessment (VIBRANT) sequence, with repetition time (TR) = 5 msec, echo time (TE) = 2 msec, flip angle (FA) =10°, field of view (FOV) = 34×34 cm^2^, matrix size = 416×416, and slice thickness = 1.2 mm. The DCE series consisted of six phases: one pre-contrast and five post-contrast. The scan time for each phase was 90 seconds. After the pre-contrast images were acquired, gadopentetic dimeglumine (Magnevist; Bayer Schering Pharma, Berlin, Germany) was intravenously injected in a 0.1 mmol/kg body weight with a flow rate of 2 ml/s, followed by 20 ml saline solution flush at the same rate.

**Ⅱ. Image acquisition of BMUS and SE**

The HI VISION Preirus system (Hitachi Medical, Tokyo, Japan) equipped with a 5-13 MHz linear transducer was used for ultrasound examinations. The BMUS was first performed with optimal Imaging parameters to visualize and detect the lesion. The greyscale image of the target lesion with the largest long axis cross-section was remained. After standard conventional BMUS, SE was performed as follows: a rectangle region of interest (ROI) was adjusted to include the target lesion and surrounding normal breast tissue, subcutaneous fat layer and pectoral muscle layer. Subsequently, the radiologist implemented perpendicularly appropriate pressure to the target lesion with the transducer. In order to improve the quality of SE image, the pressure indicator shown on the screen should read 3 to 4. The Hitachi Preirus elastography system can provide dual-modality visualization on a full screen. In SE image, the right part is a gray-scale BMUS image, and the left part is a composite of translucent color elastographic image and the corresponding BMUS image.

**Ⅲ. Post-processing of SE image**

Due to the SE elastogram was a composite color RGB image consisting of translucent color elastographic image superimposed on the gray-scale BMUS image, the elasticity data reconstruction were performed to generate a purified gray-scale elasticity image for feature extraction. The processing steps of elasticity data reconstruction have been described in detail by Zhang and Xiao et al [1,2], and can be summarized into two points: (1) the pure color elasticity image can be obtained by subtracting the original BMUS image from the composite image; (2) converting the pure color elasticity image into a gray-scale elasticity image according to the rule of color coding. The gray-scale elasticity image was used for subsequent radiomics analysis.

**Reference:**

1. Zhang Q, Xiao Y, Suo J et al (2017) Sonoelastomics for Breast Tumor Classification: A Radiomics Approach with Clustering-Based Feature Selection on Sonoelastography. Ultrasound Med Biol 43:1058-1069.

2. Xiao Y, Zeng J, Niu L et al (2014) Computer-aided diagnosis based on quantitative elastographic features with supersonic shear wave imaging. Ultrasound Med Biol 40:275-286.

**Supplementary Appendix A2** Radiomics score calculation formula

**(1) DCE-2D Rad-score =** (-13.33790207) + lbp-3D-k_glszm_GrayLevelNonUniformityNormalized * 1.46191094+

lbp-3D-k_glszm_GrayLevelVariance * (-0.73921026) +

lbp-3D-k_ngtdm_Strength * (-0.07882276) +

log-sigma-2-0-mm-3D_ngtdm_Coarseness * (-4.62517634) +

original_ngtdm_Coarseness * (-1.12887794) +

wavelet-HHL_glcm_MCC * (-0.69867290) +

wavelet-LHL_glcm_MaximumProbability * (-12.26658378) +

wavelet-LLL_glcm_Idmn * (14.05645089)

**(2) DCE-3D** **Rad-score =** (4.854310e-01) +

lbp-3D-k_glcm_ClusterShade * (5.504420e+00) +

lbp-3D-k_glszm_ZoneEntropy * (7.790739e-01) +

lbp-3D-k_glszm_ZonePercentage * (-4.773013e+01) +

lbp-3D-m1_firstorder_Kurtosis * (-1.645885e-01) +

log-sigma-3-0-mm-3D_firstorder_Uniformity * (-5.351211e-01) +

log-sigma-3-0-mm-3D_glcm_ClusterProminence * (-5.411427e-07) +

log-sigma-3-0-mm-3D_glcm_MaximumProbability * (-6.107910e+00) +

original_gldm_LargeDependenceLowGrayLevelEmphasis * (-2.102495e+01) +

original_glrlm_LongRunLowGrayLevelEmphasis * (-3.240217e+01) +

wavelet-HHH_gldm_LargeDependenceEmphasis * (4.896079e-03) +

wavelet-HLH_glcm_Imc1 * (3.908343e-01) +

wavelet-HLH_glcm_Imc2 * (-1.447918e+00) +

wavelet-HLL_firstorder_Kurtosis * (-2.502902e-01) +

wavelet-LHH_firstorder_Maximum * (8.605616e-04) +

wavelet-LHH_glcm_JointEntropy * (6.579880e-02) +

wavelet-LHH_ngtdm_Complexity * (5.121825e-05) +

wavelet-LLH_glcm_ClusterShade * (-1.236788e-05) +

wavelet-LLH_ngtdm_Busyness * (1.229334e+00)

**(3) BMUS** **Rad-score =** (-7.179209e+00) +

lbp-3D-k_glrlm_RunEntropy * (3.959424e-01)+

wavelet-HHH_gldm_SmallDependenceHighGrayLevelEmphasis * (-1.076250e+01) +

wavelet-HLL_glszm_ZonePercentage * (-2.116564e+01) +

wavelet-LHH_glcm_Imc2 * (-1.785208e+00) +

wavelet-LHL_firstorder_Mean * (-1.878513e+00) +

wavelet-LHL_glszm_ZoneEntropy * (4.816197e-04) +

wavelet-LLH_firstorder_Skewness * (4.985084e-01) +

wavelet-LLL_glcm_Idn * (7.673394e+00)

**(4) SE** **Rad-score =** (-4.973768713) +

lbp-3D-k_glrlm_RunPercentage * (-9.285848032) +

original_firstorder_Maximum * (0.001599095) +

original_firstorder_Mean * (0.027134562) +

original_shape_Maximum2DDiameterRow * (0.004060973) +

wavelet-LHL_firstorder_Mean * (-2.049758331) +

wavelet-LLL_firstorder_Mean * (0.011198967) +

wavelet-LLL_ngtdm_Coarseness * (-43.906307948)

**(5) BMUS+SE** **Rad-score =** (-44.771503) +

SE_lbp-3D-k_glrlm_RunPercentage * (16.614429) +

SE_original_firstorder_Maximum * (-0.007288) +

SE_original_firstorder_Mean * (-6.076021) +

SE_original_shape_Maximum2DDiameterRow * (-0.010946) +

SE_wavelet-LHL_firstorder_Mean * (-11.213982) +

SE_wavelet-LLL_firstorder_Mean * (2.176813) +

SE_wavelet-LLL_ngtdm_Coarseness * (-40.934157) +

BMUS_lbp-3D-k_glrlm_RunEntropy * (1.258024) +

BMUS_wavelet-HHH_gldm_SmallDependenceHighGrayLevelEmphasis*(-22.278966)+

BMUS_wavelet-HLL_glszm_ZonePercentage * (18.567977) +

BMUS_wavelet-LHH_glcm_Imc2 * (-5.877999) +

BMUS_wavelet-LHL_firstorder_Mean * (-2.473586) +

BMUS_wavelet-LHL_glszm_ZoneEntropy * (0.320862) +

BMUS_wavelet-LLH_firstorder_Skewness * (0.882354) +

BMUS_wavelet-LLL_glcm_Idn * (36.372949)

**(6) DCE-3D+BMUS** **Rad-score =** (-5.753e+01) +

DCE-3D_lbp-3D-k_glcm_ClusterShade * (2.048e+01) +

DCE-3D_lbp-3D-k_glszm_ZoneEntropy * (6.554e-01) +

DCE-3D_lbp-3D-k_glszm_ZonePercentage * (-8.842e+01) +

DCE-3D_lbp-3D-m1_firstorder_Kurtosis * (-4.310e-01) +

DCE-3D_log-sigma-3-0-mm-3D_firstorder_Uniformity * (-3.470e+01) +

DCE-3D_log-sigma-3-0-mm-3D_glcm_ClusterProminence * (-3.024e-06) +

DCE-3D_log-sigma-3-0-mm-3D_glcm_MaximumProbability * (1.427e+01) +

DCE-3D_original_gldm_LargeDependenceLowGrayLevelEmphasis * (-1.184e+02) +

DCE-3D_original_glrlm_LongRunLowGrayLevelEmphasis * (1.747e+02) +

DCE-3D_wavelet-HHH_gldm_LargeDependenceEmphasis * (1.272e-01) +

DCE-3D_wavelet-HLH_glcm_Imc1 * (1.369e+01) +

DCE-3D_wavelet-HLH_glcm_Imc2 * (9.461e+00) +

DCE-3D_wavelet-HLL_firstorder_Kurtosis * (-1.258e+00) +

DCE-3D_wavelet-LHH_firstorder_Maximum * (9.756e-04) +

DCE-3D_wavelet-LHH_glcm_JointEntropy * (6.054e-01) +

DCE-3D_wavelet-LHH_ngtdm_Complexity * (5.518e-04) +

DCE-3D_wavelet-LLH_glcm_ClusterShade * (-1.734e-04) +

DCE-3D_wavelet-LLH_ngtdm_Busyness * (5.314e+00) +

BMUS_lbp-3D-k_glrlm_RunEntropy * (-4.792e-01) +

BMUS_wavelet-HHH_gldm_SmallDependenceHighGrayLevelEmphasis*(-1.549e+01)+

BMUS_wavelet-HLL_glszm_ZonePercentage * (-6.158e+00) +

BMUS_wavelet-LHH_glcm_Imc2 * (-6.045e+00) +

BMUS_wavelet-LHL_firstorder_Mean * (-2.756e+00) +

BMUS_wavelet-LHL_glszm_ZoneEntropy * (3.771e-01) +

BMUS_wavelet-LLH_firstorder_Skewness * (8.168e-01) +

BMUS_wavelet-LLL_glcm_Idn * (5.566e+01)

**(7) DCE-3D+SE** **Rad-score =** (-1.148e+01) +

DCE-3D_lbp-3D-k_glcm_ClusterShade * (1.796e+01) +

DCE-3D_lbp-3D-k_glszm_ZoneEntropy * (7.718e-01) +

DCE-3D_lbp-3D-k_glszm_ZonePercentage * (-8.103e+01) +

DCE-3D_lbp-3D-m1_firstorder_Kurtosis * (-8.316e-01) +

DCE-3D_log-sigma-3-0-mm-3D_firstorder_Uniformity * (-5.513e+01) +

DCE-3D_log-sigma-3-0-mm-3D_glcm_ClusterProminence * (-3.062e-06) +

DCE-3D_log-sigma-3-0-mm-3D_glcm_MaximumProbability * (2.995e+01) +

DCE-3D_original_gldm_LargeDependenceLowGrayLevelEmphasis * (-1.166e+02) +

DCE-3D_original_glrlm_LongRunLowGrayLevelEmphasis * (2.797e+02) +

DCE-3D_wavelet-HHH_gldm_LargeDependenceEmphasis * (9.600e-02) +

DCE-3D_wavelet-HLH_glcm_Imc1 * (2.820e+01) +

DCE-3D_wavelet-HLH_glcm_Imc2 * (1.563e+01) +

DCE-3D_wavelet-HLL_firstorder_Kurtosis * (-1.142e+00) +

DCE-3D_wavelet-LHH_firstorder_Maximum * (2.845e-03) +

DCE-3D_wavelet-LHH_glcm_JointEntropy * (-6.625e-03) +

DCE-3D_wavelet-LHH_ngtdm_Complexity * (6.673e-04) +

DCE-3D_wavelet-LLH_glcm_ClusterShade * (-1.685e-04) +

DCE-3D_wavelet-LLH_ngtdm_Busyness * (6.969e+00) +

SE_lbp-3D-k_glrlm_RunPercentage * (-4.908e+01) +

SE_original_firstorder_Maximum * (-1.022e-02) +

SE_original_firstorder_Mean * (-4.936e+00) +

SE_original_shape_Maximum2DDiameterRow * (5.975e-03) +

SE_wavelet-LHL_firstorder_Mean * (-9.485e+00) +

SE_wavelet-LLL_firstorder_Mean * (1.777e+00) +

SE_wavelet-LLL_ngtdm_Coarseness * (-3.185e+00)

**(8) All-Combination** **Rad-score =** (-5.890e+01) +

DCE-3D_lbp-3D-k_glcm_ClusterShade * (1.764e+01) +

DCE-3D_lbp-3D-k_glszm_ZoneEntropy * (5.703e-01) +

DCE-3D_lbp-3D-k_glszm_ZonePercentage * (-1.166e+02) +

DCE-3D_lbp-3D-m1_firstorder_Kurtosis * (-9.036e-01) +

DCE-3D_log-sigma-3-0-mm-3D_firstorder_Uniformity * (-5.744e+01) +

DCE-3D_log-sigma-3-0-mm-3D_glcm_ClusterProminence * (-3.626e-06) +

DCE-3D_log-sigma-3-0-mm-3D_glcm_MaximumProbability * (3.348e+01) +

DCE-3D_original_gldm_LargeDependenceLowGrayLevelEmphasis * (-1.180e+02) +

DCE-3D_original_glrlm_LongRunLowGrayLevelEmphasis * (3.202e+02) +

DCE-3D_wavelet-HHH_gldm_LargeDependenceEmphasis * (1.132e-01) +

DCE-3D_wavelet-HLH_glcm_Imc1 * (3.293e+01) +

DCE-3D_wavelet-HLH_glcm_Imc2 * (1.801e+01) +

DCE-3D_wavelet-HLL_firstorder_Kurtosis * (-1.265e+00) +

DCE-3D_wavelet-LHH_firstorder_Maximum * (3.633e-03) +

DCE-3D_wavelet-LHH_glcm_JointEntropy * (1.744e-01) +

DCE-3D_wavelet-LHH_ngtdm_Complexity * (7.247e-04) +

DCE-3D_wavelet-LLH_glcm_ClusterShade * (-1.434e-04) +

DCE-3D_wavelet-LLH_ngtdm_Busyness * (7.360e+00) +

SE_lbp-3D-k_glrlm_RunPercentage * (-3.679e+01) +

SE_original_firstorder_Maximum * (-4.076e-02) +

SE_original_firstorder_Mean * (-1.127e+01) +

SE_original_shape_Maximum2DDiameterRow * (-1.489e-02) +

SE_wavelet-LHL_firstorder_Mean * (-1.590e+01) +

SE_wavelet-LLL_firstorder_Mean * (4.019e+00) +

SE_wavelet-LLL_ngtdm_Coarseness * (1.335e+02) +

BMUS_lbp-3D-k_glrlm_RunEntropy * (1.555e+00) +

BMUS_wavelet-HHH_gldm_SmallDependenceHighGrayLevelEmphasis*(5.194e+01) +

BMUS_wavelet-HLL_glszm_ZonePercentage * (-2.058e+02) +

BMUS_wavelet-LHH_glcm_Imc2 * (-4.199e+00) +

BMUS_wavelet-LHL_firstorder_Mean * (-2.485e+00) +

BMUS_wavelet-LHL_glszm_ZoneEntropy * (7.899e-01) +

BMUS_wavelet-LLH_firstorder_Skewness * (9.931e-01) +

BMUS_wavelet-LLL_glcm_Idn * (3.483e+01)

**Supplementary Figure S1** Radiomics feature selection using the least absolute shrinkage and selection operator (LASSO) logistic regression in the DCE-2D **(A, B)**, DCE-3D **(C, D)**, BMUS **(E, F)** and SE **(G, H)** radiomics signatures, respectively. **(A, C, E, G)** Tuning parameter (λ) in the LASSO logistic model used 10-fold cross-validation were followed. The area under the curve (AUC) was plotted versus log (λ). **(B, D, F, H)** LASSO coefficient profiles of the selected features from each radiomics signature.


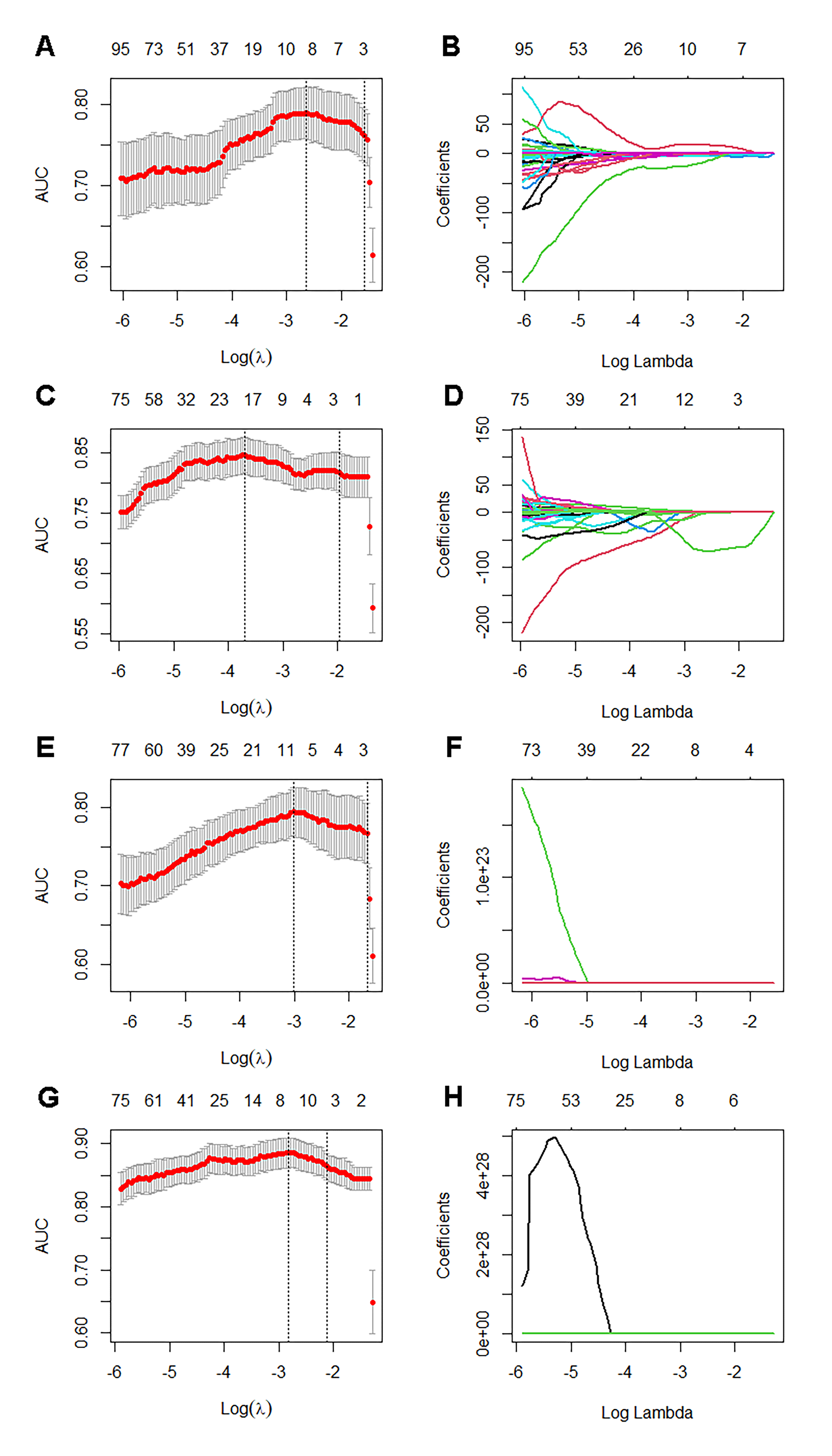


**Supplementary Figure S2** Receiver operating characteristic (ROC) curves of the single modality radiomics signatures in the training (A) and testing (B) datasets.


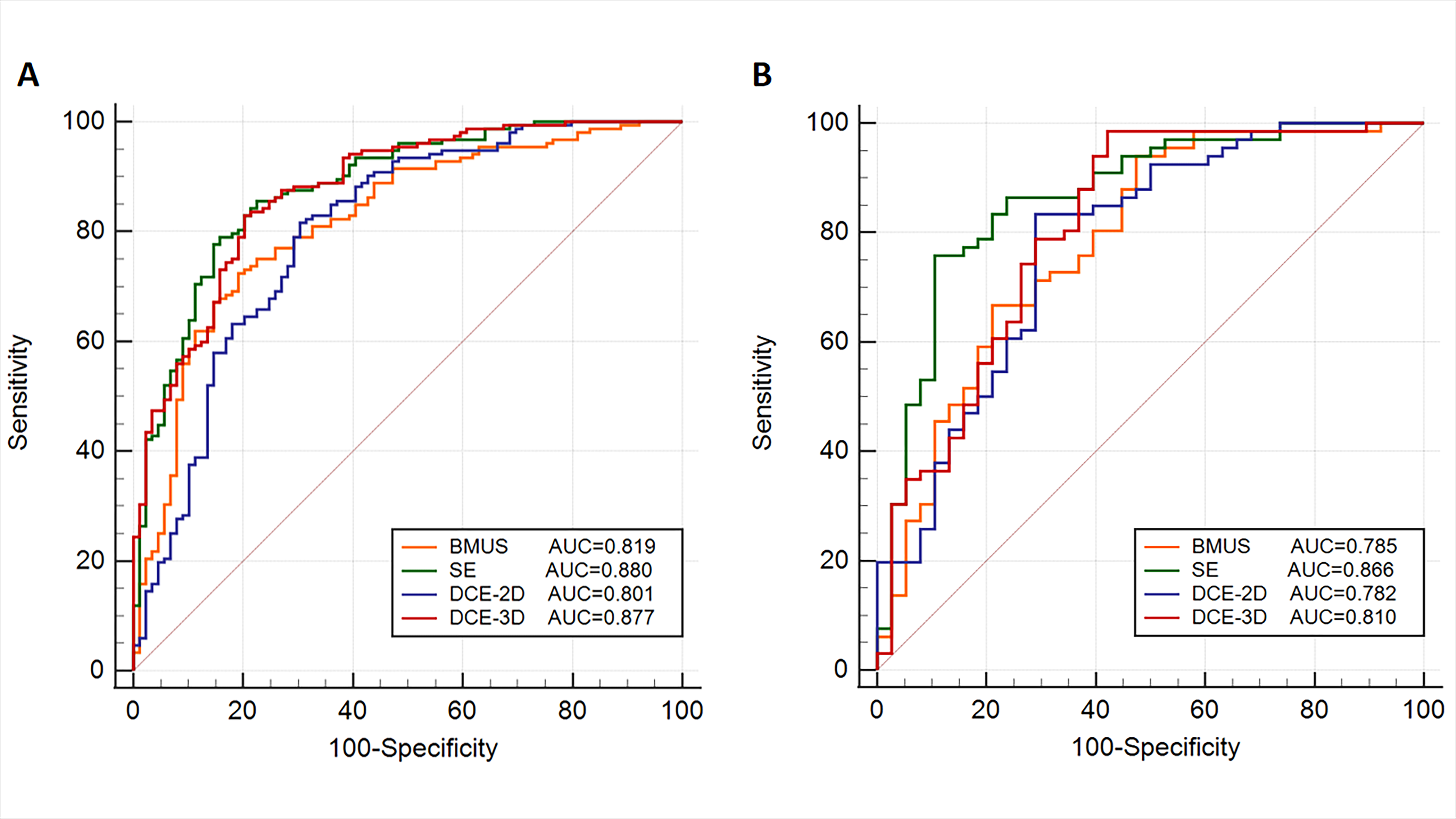


**Supplementary Figure S3** Receiver operating characteristic (ROC) curves of the multi-

modality radiomics signatures in the training (A) and testing (B) datasets.

**
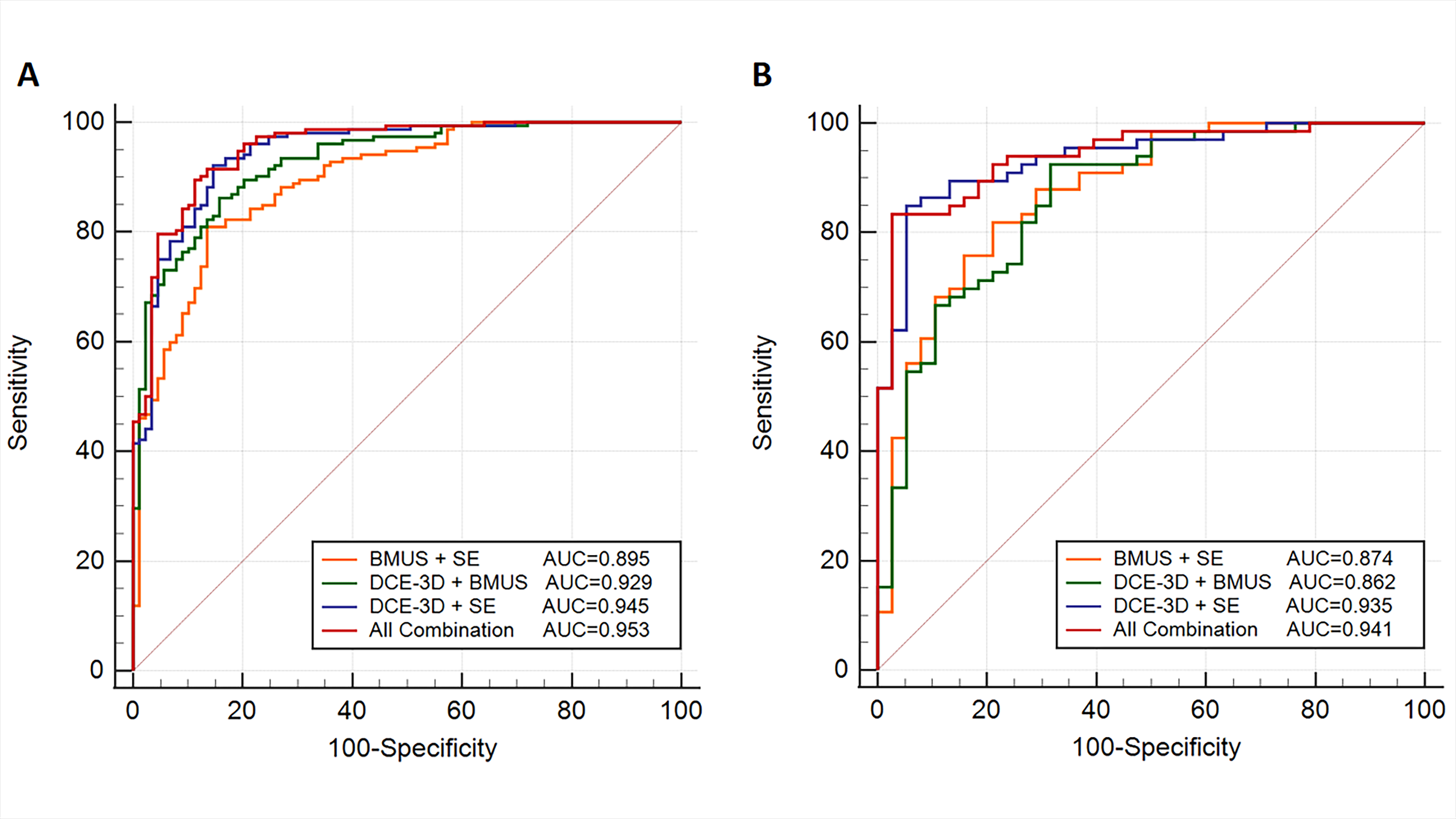
**

**Supplementary Figure S4** Waterfall plot showing Rad-score for each lesion of the All-Combination radiomics signature in the training (A) and testing (B) datasets with the cutoff value of 0.797

**
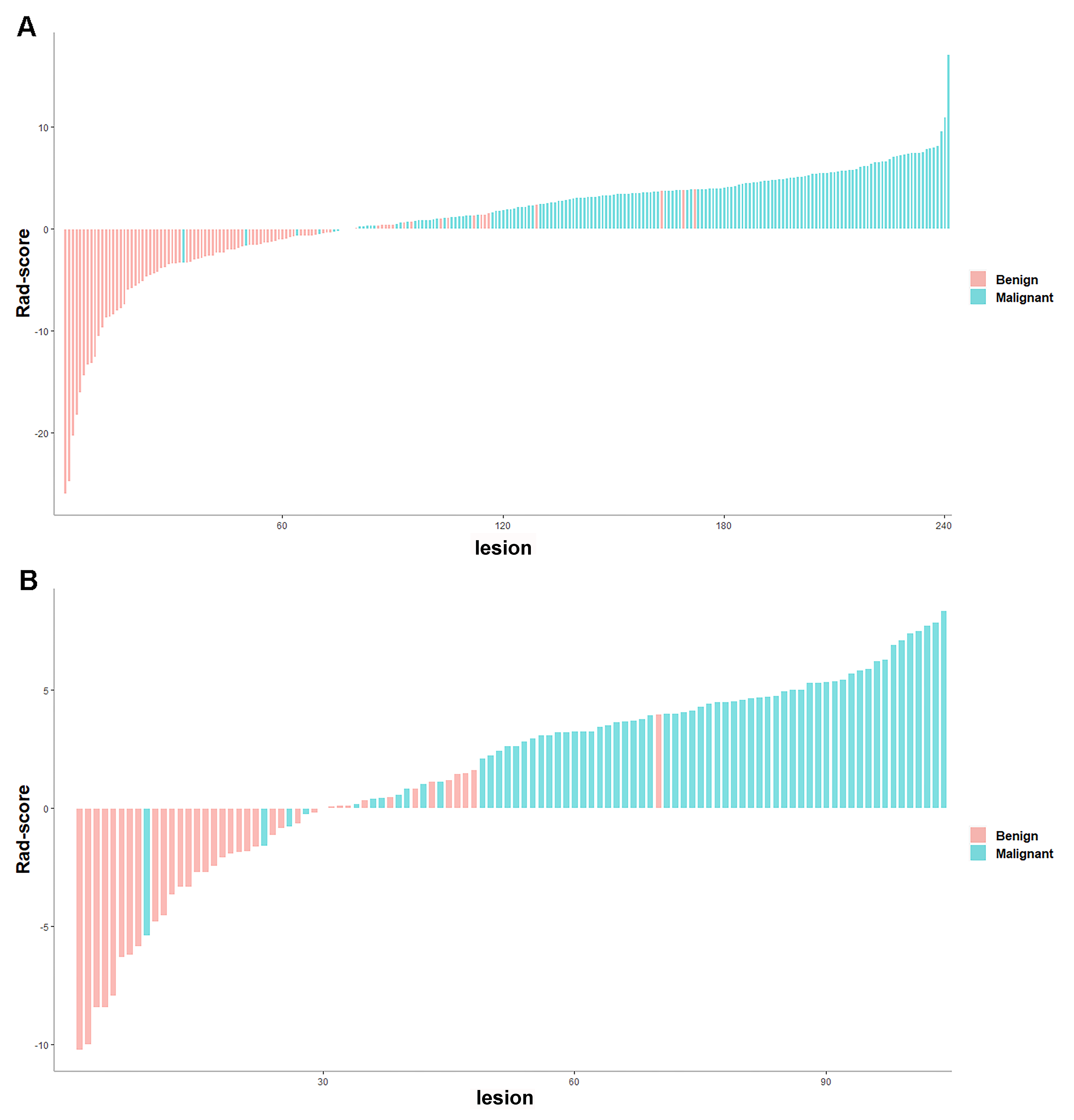
**

**Supplementary Table S1** Baseline demographic and characteristics of breast lesions from corresponding patients in the training and testing datasets

| **Characteristic** | **Training dataset**  **(n = 241)** | **Testing dataset**  **(n = 104)** | ***p* value** |
| --- | --- | --- | --- |
| Age (years) |  |  |  |
| Mean ± SD | 47.8 ± 9.9 | 48.1 ± 10.7 | 0.84 |
| Range | 22-78 | 23-72 |  |
| Maximal diameter (cm) |  |  |  |
| Mean ± SD | 2.0 ± 1.3 | 2.3 ± 1.3 | 0.08 |
| Range | 0.3-7.8 | 0.5-6.5 |  |
| Primary site |  |  | 0.22 |
| Left | 110 (45.6%) | 55 (52.9%) |  |
| Right | 131 (54.4%) | 49 (47.1%) |  |
| BI-RADS category |  |  | 0.39 |
| 2 - 4A | 88 (36.5%) | 33 (31.7%) |  |
| 4B - 5 | 153 (63.5%) | 71 (68.3%) |  |

Abbreviation: BI-RADS, Breast Imaging Reporting and Data System.

**Supplementary Table S2** Pathological types of breast lesions in the training and testing datasets

| **Pathological results** | **Training dataset**  **(N = 241)** | **Testing dataset**  **(N = 104)** |
| --- | --- | --- |
| **Benign** | **89 (36.9%)** | **38 (36.5%)** |
| Fibroadenoma | 27 (11.2%) | 9 (8.7%) |
| Adenosis | 39 (16.2%) | 16 (15.4%) |
| Intraductal papilloma | 11 (4.6%) | 6 (5.8%) |
| Inflammation | 6 (2.5%) | 7 (6.7%) |
| others | 6 (2.5%) | 0 (0.0%) |
| **Malignant** | **152 (63.1%)** | **66 (63.5%)** |
| Invasive ductal carcinoma | 116 (48.1%) | 52 (50.0%) |
| Ductal carcinoma in situ | 25 (10.4%) | 6 (5.8%) |
| Invasive lobular carcinoma | 3 (1.2%) | 4 (3.8%) |
| Mucinous carcinoma | 3 (1.2%) | 1 (1.0%) |
| others | 5 (2.1%) | 3 (2.9%) |

**Supplementary Table S3** Comparison of AUCs between the radiomics nomogram, radiomics signatures and BI-RADS classification

|  | **Training dataset** | | |  | **Testing dataset** | | |
| --- | --- | --- | --- | --- | --- | --- | --- |
|  | **AUC** | **95% CI** | ***p* value^#^** |  | **AUC** | **95% CI** | ***p* value^#^** |
| Radiomics nomogram | 0.964 | 0.932 - 0.984 | Reference |  | 0.951 | 0.890 - 0.983 | Reference |
| DCE-2D | 0.801 | 0.745 - 0.850 | <0.001 |  | 0.782 | 0.690 - 0.857 | <0.001 |
| DCE-3D | 0.877 | 0.828 - 0.915 | <0.001 |  | 0.810 | 0.722 - 0.880 | 0.002 |
| SE | 0.880 | 0.832 - 0.918 | <0.001 |  | 0.866 | 0.785 - 0.925 | 0.03 |
| BMUS | 0.819 | 0.765 - 0.866 | <0.001 |  | 0.785 | 0.693 - 0.859 | 0.001 |
| All-Combination | 0.953 | 0.918 - 0.976 | 0.052 |  | 0.941 | 0.877 - 0.978 | 0.33 |
| BI-RADS | 0.910 | 0.867 - 0.943 | 0.01 |  | 0.909 | 0.837 - 0.957 | 0.16 |

^#^By Delong test.

Abbreviation: BI-RADS, Breast Imaging Reporting and Data System.
